# Study on the Influence Mechanism of Core–Shell Emulsion Admixture on Rheological Properties of Cement Mortar

**DOI:** 10.3390/ma19132733

**Published:** 2026-06-25

**Authors:** Shuncheng Xiang, Rui Wang, Jie Chen, Xubiao Luo, Huan Zhou, Xin Yang, Yuelin Li, Jing Zhang, Zhen Jiang, Zheng Len, Yanqi He, Yang Liu

**Affiliations:** 1 Hunan Provincial Engineering Technology Research Center for Novel and Carbon Neutral Road Material, Changsha University of Science & Technology, Changsha 410114, China; 2 School of Hydraulic and Ocean Engineering, Changsha University of Science & Technology, Changsha 410114, China; 3 Key Laboratory of Jiangxi Province for Functional Biology and Pollution Control in Red Soil Regions, School of Life Sciences, Jinggangshan University, Ji’an 343009, China; 4 Science and Technology Affairs Center of Hunan Province, Changsha 410082, China; 5 China Construction West Hunan Co., Ltd., Changsha 410000, China

**Keywords:** core–shell structured emulsion, cement mortar, rheological properties, regulation mechanism

## Abstract

Traditional research was mostly focused on the effects of emulsions on the mechanical properties and durability of cement mortar, while studies on the regulation mechanism of emulsions on the rheological properties of cement-based materials and the coupling mechanism with the hydration process were rarely conducted. In this paper, a novel core–shell structured emulsion was prepared by free radical polymerization. The regulation of cement mortar yield stress, creep recovery, dynamic viscosity, and thixotropy by different dosages (0–10%) of the emulsion admixture was systematically investigated, and combined with characterization by scanning electron microscopy (SEM), X-ray diffraction (XRD), and Fourier transform infrared spectroscopy (FTIR), the microscopic action mechanism of the emulsion was elucidated. It was demonstrated that the Bingham fluid behavior of cement mortar was not altered by the core–shell emulsion, whereas a significant dosage-dependent regulatory effect on its rheological parameters was observed, and a critical regulation interval of 4–6% was identified. At an emulsion dosage of 10%, the yield stress of the mortar was increased by 937.0% compared to that of the control group. At dosages of 2–4%, the static structural stability and construction flowability of the mortar were synergistically optimized, and the weakest thixotropy and the best structural stability were exhibited at an emulsion dosage of 4%. A more pronounced shear-thinning behavior was shown by all modified mortars, and their high-shear flowability was not affected. Microstructural analysis confirmed that no chemical reaction occurred between the emulsion and the cement hydration products. Through the triple effects of “hydration retardation by physical coating, pore filling and densification, and composite network enhancement”, a film was formed on the surface of cement particles by the emulsion, which hindered the diffusion of water and ions, thereby regulating the cement hydration process and microstructural evolution.

## 1. Introduction

The key roles of lubrication and filling were found to be played by the emulsion admixture in cement mortar. The pore solution surface tension was effectively reduced, the flowability of the paste was improved, and the workability was enhanced [1]. Additionally, a polymer film was formed within the interior, the bond between cement stone and aggregate was enhanced, and the compressive strength, flexural strength, and toughness were improved [2]. Moreover, the internal pores and capillary channels in the mortar were effectively blocked by the polymer film, and the resistance to seepage, frost, and chemical erosion was enhanced, thereby effectively improving the durability of cement mortar [3,4]. In traditional studies, research was mainly focused on the effects of emulsion admixtures on the mechanical properties and durability of cement mortar, while studies on the regulation and optimization of the rheological properties of cement paste were relatively limited. It was found by Xuesong Han [5] that, with a 20% dosage of hydrophobic polyacrylate emulsion, the interfacial bond strength and flexural toughness of underwater epoxy-cement mortar were optimized, while the flexural and compressive strengths were maintained. It was demonstrated by Hyung-Jun Kim [6] that the compressive strength and elastic modulus of cement mortar were reduced at different temperatures with the addition of ethylene-vinyl acetate polymer powder. The feasibility of using polyethylene terephthalate polymer cement mortar in reinforced concrete structures was demonstrated by A.S. Pakdel [7]. The interfacial conditions and water absorption properties of polymer-modified cement mortar used for repairing reinforced concrete structures eroded by salt were explored by Wang Yong [8].

**Figure 1:**
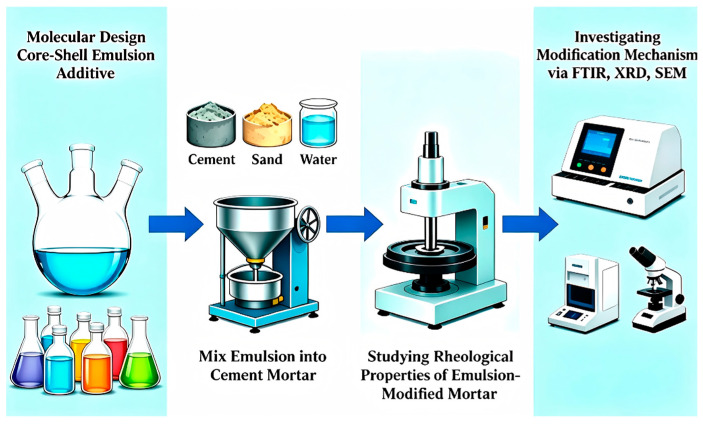
Overall Technical Flow Chart.

In the above-mentioned studies, the focus was mainly placed on the influence patterns of emulsions on the mechanical properties and durability of cement mortar. However, studies on the regulation mechanism of emulsions on the rheological properties of cement-based materials and their coupling effect with the hydration process were relatively limited, and a systematic correlation analysis between the evolution law of rheological parameters and the microscopic action mechanism was lacking. For the purpose of regulating the construction performance of high-performance cement-based materials and promoting the application of core–shell emulsions in construction engineering, the influence of the core–shell structured emulsion on the rheological properties of cement mortar was investigated in this paper. Combined with macro- and micro-experimental analyses, the regulation mechanism of the core–shell structured emulsion on the rheological properties of cement mortar was eventually obtained, and the correspondence between rheological parameters and microstructural evolution was established. The overall research technical flowchart of this paper is shown in Figure 1 below. This figure comprehensively presents the entire research process of “molecular design and synthesis of core–shell emulsion–preparation of cement mortar specimens–macroscopic rheological property testing–microstructural and phase characterization.”

## 2. Materials and Methods

### 2.1. Materials

#### 2.1.1. Cement

The cement used in this study was produced by Yangchun Building Materials Co., Ltd. (Weifang, China), and its grade was determined to be P·O 42.5. The test indices were based on the GB175-2020 standard for common Portland cement [9]. The chemical and mineral compositions of the cement were determined using an X-ray fluorescence spectrometer (S8 Tiger, Bruker Corporation, Bremen, Germany). The relevant chemical and mineral compositions were presented in Table 1, and the physical properties were presented in Table 2.

#### 2.1.2. Fine Aggregate

The fine aggregate used was medium sand produced in China West Construction Hunan Co., Ltd., Changsha, China, which was confirmed to comply with the GB/T 14684-2022 standard [10]. Its fineness modulus was determined to range from 2.6 to 3.0, and its clay content was found to be less than 1.0%. The sieving results of the fine aggregate were presented in Table 3.

#### 2.1.3. Water

The water used for the experiments was tap water from Changsha City, China, which was confirmed to comply with the JGJ 63-2006 specification [11].

### 2.2. Synthesis of Core–Shell Structured Emulsion

**Figure 2:**
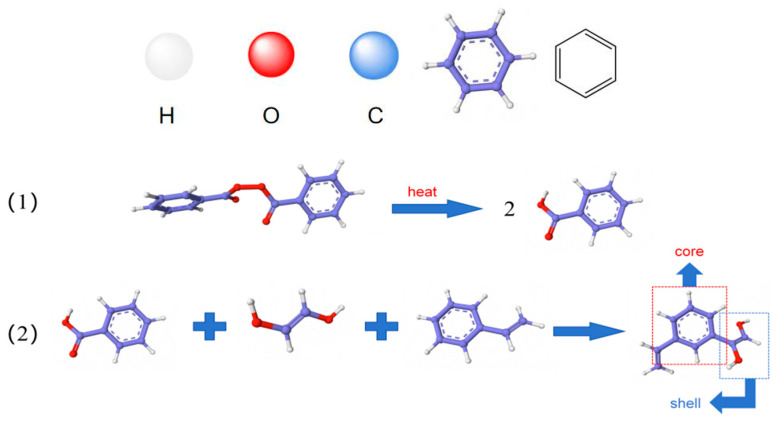
Schematic diagram of prepolymer reaction and molecular structure of core–shell emulsifier.

① Synthesis of core–shell structured prepolymer: This stage was implemented as a seeded prepolymerization process, the core objective of which was that an emulsifier prepolymer bearing hydrophilic active groups was prepared, and seed latex as well as a stable emulsifying system were provided for the subsequent synthesis of core-shell structured emulsion. Styrene, butyl acrylate, and methyl methacrylate were used as monomers, and benzoyl peroxide (BPO) and potassium persulfate were employed as composite initiators. As a water-soluble initiator, a stable decomposition rate, strong controllability of polymerization reaction, superior compatibility with aqueous polymerization system, and prominent performance in significantly improving the storage stability of emulsion were exhibited by potassium persulfate; in addition, a synergistic initiation effect was formed between potassium persulfate and the oil-soluble initiator BPO. A prepolymer with a hydrophobic core layer and a shell layer containing hydrophilic active groups was successfully prepared, and the specific experimental procedures were as follows:

Styrene, butyl acrylate, and methyl methacrylate were poured into a three-necked flask at a monomer ratio of 3:2:1. Benzoyl peroxide (BPO) and potassium persulfate were added at 1% and 0.8% of the total monomer mass, respectively, and free radical polymerization was carried out with polyoxyethylene alcohol. The emulsifier prepolymer was thereby obtained, as shown in Figure 2.

② Synthesis of core–shell structured emulsion admixture: This stage was carried out as the final polymerization and formation process of the emulsion, the prepolymer prepared in Step 1 was used as both the emulsifier and the seed latex, the semi-continuous titration method was adopted, and free radical polymerization was initiated by potassium persulfate in an aqueous phase environment, through which monomers were continuously polymerized on the surface of the seed latex, and the finished core–shell structured emulsion product with stable structure and controllable performance was obtained. The specific experimental procedures were as follows:

Styrene, butyl acrylate, the emulsifier prepolymer, and potassium persulfate were used as monomer raw materials. Component A and Component B solutions were prepared for later use.

Component A: Styrene and butyl acrylate.

Component B: Emulsifier prepolymer, potassium persulfate, and deionized water.

**Figure 3:**
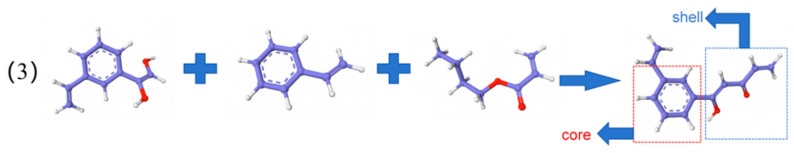
Schematic diagram of reaction and molecular structure of core–shell structure emulsion admixture.

**Figure 4:**
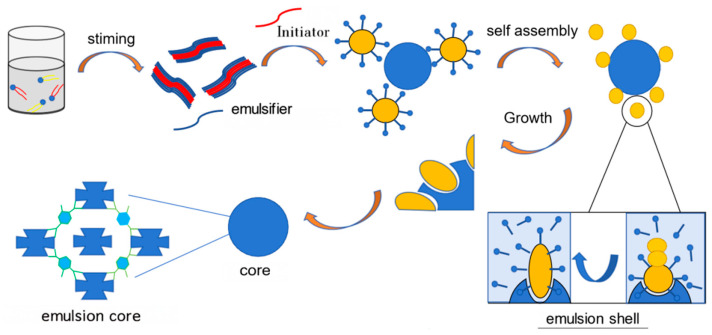
Schematic diagram of free radical polymerization mechanism of core–shell emulsion admixture.

Component A was placed at the bottom of a three-necked flask. Component B was titrated into the flask through a long-neck funnel over a period of 2 h. During the titration process, the temperature was controlled and maintained between 60 °C and 80 °C to prevent emulsion breaking. The pH was adjusted to 7–8 using sodium hydroxide, resulting in the formation of the core–shell structured emulsion. The relevant reaction principles and the structure of the obtained core–shell emulsion were illustrated in Figure 3 and Figure 4.

### 2.3. Mix Design

According to the national standard JTG 3420-2020 [12], the mixing proportions of the mortar with the emulsion were presented in Table 4, in which the water-to-cement ratio was fixed at 50 wt.%, the cement-to-sand ratio was set at 1:3 (with the sand content being 300 wt.% of the cement mass), and the solid dosages of the emulsion, calculated by the mass of cement, were 0, 2, 4, 6, 8, and 10 wt.%.

### 2.4. Sample Preparation and Curing

Cement and sand were added into a standard cement mortar mixer (JJ-5, Wuxi Jianyi Instrument Co., Ltd., Wuxi, China) and mixed at 140 rpm for 3 min to ensure thorough mixing of the materials. Water containing the core–shell structured emulsion was added and mixed at 140 rpm for 3 min, resulting in a uniformly distributed paste. Immediately after mixing, the mortar was loaded into a dedicated sample cup of the rheometer, ensuring that the distance between the paste surface and the cup rim was no less than 40 mm to minimize wall effects. Subsequently, the mortar temperature was stabilized at 20 °C using the built-in temperature control system of the rheometer, and then the test was conducted. The remaining freshly mixed mortar was poured into prismatic molds of 40 mm × 40 mm × 160 mm, allowed to cure naturally for 24 h, and then demolded. Afterward, the specimens were placed in a standard curing room (20 ± 2 °C, humidity ≥ 95%) and cured until the specified ages for subsequent microstructural testing.

### 2.5. Test Methods

#### 2.5.1. Rheological Property Testing

1. Test Apparatus

The rheological properties of cement mortar were measured using an Anton Paar MCR 302 rotational rheometer (Anton Paar GmbH, Graz, Austria), which was equipped with a parallel-plate rotor (φ50 mm). The gap between the plates was fixed at 2 mm, and the temperature was maintained at 20 ± 0.5 °C throughout the test to eliminate interference from temperature variations on the test results. The testing procedure was carried out in accordance with T/CECS 10511-2025 “Coaxial Rotor Test Method for Rheological Properties of Fresh Cement Paste and Mortar” [13].

2. Test Scheme and Core Parameters
  (1) Steady-state shear test (characterization of yield stress and shear-thinning behavior)

A continuous ramp-up steady-state shear mode was employed. The shear rate was increased within a range of 0–100 s^−1^, and 30 measurement points were set at logarithmic intervals. An equilibration time of 3s was allowed at each point. After the data stabilized, the changes in shear stress and dynamic viscosity with shear rate were recorded.

  (2) Cyclic shear test (characterization of thixotropy)

A ramp-up/ramp-down cyclic shear mode was used, consisting of two consecutive stages: ① Ramp-up stage: the shear rate was linearly increased from 0 to 200 s^−1^ over 60 s; ② Ramp-down stage: the shear rate was linearly decreased from 200 to 0 s^−1^ over 60 s. Thirty measurement points were equally spaced in both the ramp-up and ramp-down stages, and the relationship between shear stress and shear rate was recorded throughout the whole process.

  (3) Creep and recovery test

A constant-stress creep mode was applied with a loading stress of 200 Pa and a duration of 100 s. A sampling interval of 0.1 s was used, and the variation in the sample deflection angle with time was recorded to characterize the creep deformation resistance of the paste.

3. Quantitative calculation method for rheological parameters
  (1) Yield stress

It was calculated by fitting the Bingham model commonly used for cement-based materials. The model expression was given as follows:

(1) $${\tau \;=\;\tau }_{0}\;+\;\eta_{\mathrm{pl}}\gamma$$

where τ was defined as the shear stress (Pa); $\tau_{0}$ was defined as the yield stress (Pa), i.e., the critical shear stress at which the paste began to undergo plastic flow; $\eta_{\mathrm{pl}}$ₗ was defined as the plastic viscosity (Pa·s); and γ was defined as the shear rate (s^−1^).

  (2) Shear-thinning behavior

The shear-thinning behavior was quantitatively characterized by the reduction in dynamic viscosity, and the formula was expressed as follows:

(2) $$\eta \;=\;\frac{\tau }{\Upsilon }$$

where η was defined as the dynamic viscosity (Pa·s); τ was defined as the shear stress (Pa) at the corresponding shear rate; and Υ was defined as the shear rate (s^−1^).

The calculation formula for the viscosity reduction due to shear-thinning was given as follows:

(3) $$△\eta \;=\;\frac{\eta_{0}\;-{\;\eta }_{\infty }}{\eta_{0}}$$

where △η was defined as the reduction rate of dynamic viscosity (%), with a larger value indicating more pronounced shear-thinning behavior; $\eta_{0}$ was defined as the initial dynamic viscosity in the low-shear region; and $\eta_{\infty }$ was defined as the steady-state dynamic viscosity in the high-shear region.

  (3) Thixotropy. It was quantitatively characterized by the thixotropic hysteresis loop area, which was calculated using the shoelace formula. The formula was as follows:

(4) $$s=\frac{1}{2}|\sum_{i=1}^{n}\left(x_{i}y_{i+1}-{\;x}_{i+1}y_{i}\right)|$$

where $x_{i}$ was defined as the shear rate, and $y_{i}$ was defined as the shear stress.

  (4) Creep recovery characteristics

Three core indicators were used to quantitatively characterize the creep recovery behavior: ① the total creep deformation (the deflection angle of the paste at the end of 100 s loading, denoted as $\theta_{\mathrm{total}}$); ② the creep deformation increment (${\Delta \theta \;=\;\theta }_{100s\;}-\;\theta_{0s}$), where a smaller value indicated better creep resistance; and ③ the fast creep rate ($k\;=\;\frac{{\Delta \theta }_{0–30s}}{30}$), which was used to characterize the sensitivity of the paste to instantaneous shear deformation.

#### 2.5.2. X-Ray Diffraction (XRD)

A D8 ADVANCE X-ray diffractometer (Bruker Corporation, Bremen, Germany) was used for the test, which was performed with a Cu-Kα radiation source (λ = 0.15406 nm) at a tube voltage of 40 kV and a tube current of 40 mA. The scanning range was set from 2θ = 5° to 70°, with a step size of 0.02° and a dwell time of 0.2 s per step. Blocks taken from the central part of the cement mortar specimens at the age of 28 days were ground to 200–300 mesh in an agate mortar and were then dried. An appropriate amount of the powder sample was placed into the groove in the center of a glass sample holder using a spatula. The sample was gently pressed flat with a glass slide, and the excess powder protruding above the holder surface was scraped off to ensure a smooth surface. The test was then performed.

#### 2.5.3. Fourier Transform Infrared Spectroscopy (FTIR)

A Bruker TENSOR 27 Fourier Transform Infrared Spectrometer (Bruker Corporation, Bremen, Germany) was employed for the test, which was conducted in attenuated total reflection (ATR) mode, with the wavenumber range set from 4000 to 400 cm^−1^, a spectral resolution of 4 cm^−1^, and 32 scans per sample. Blocks taken from the central part of the cement mortar specimens at the age of 28 days were ground to 200–300 mesh in an agate mortar and were then dried. An appropriate amount of the powder sample was placed with a spatula in the center of the instrument’s glass slide. The probe was then lowered, and the test was performed.

#### 2.5.4. Scanning Electron Microscopy (SEM)

A TESCAN field-emission scanning electron microscope of the model MIRA3 LMH EDS:One Max 20 (TESCAN Orsay Holding, TESCAN, Brno, Czech Republic) was used. The cement mortar samples cured under standard conditions were maintained until the specified ages. After the reaction was terminated by immersion in anhydrous ethanol, the samples were broken into pieces, and test blocks of appropriate size were taken from the central part. They were dried in an oven at 60 °C for 12 h, and then sputter-coated with gold before SEM observation.

#### 2.5.5. AI-Assisted Content Optimization

During the preparation of this study, the authors used Doubao 2.1 for the purposes of supplementing and optimizing the schematic diagram of the overall technical flowchart (Figure 1). The authors have reviewed and edited the output and take full responsibility for the content of this publication.

## 3. Results and Discussion

### 3.1. Rheological Properties

#### 3.1.1. Yield Stress

**Figure 5:**
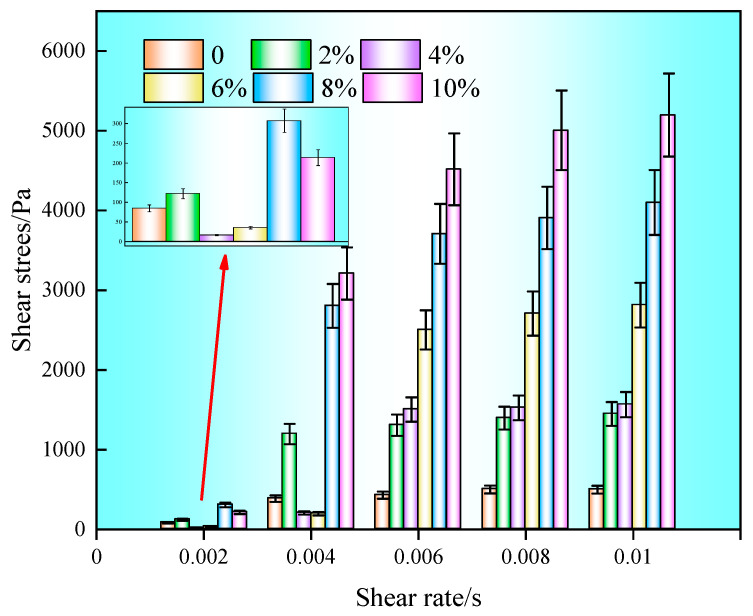
Shear rate vs. shear stress of cement mortar with different emulsion dosages.

A logarithmic ramp-up steady-state shear mode was employed for the yield stress measurement, with the shear rate range set from 0 to 100 s^−1^ and emphasis placed on the low-to-medium shear rate region. This range was selected to enable accurate fitting with the Bingham model, from which both the yield stress and plastic viscosity were calculated. These parameters were used to characterize the static structural strength and the incipient flow behavior of the paste. As can be seen from Figure 5, two distinct rheological stages were clearly distinguished for both the control group and all emulsion-modified mortars. The nonlinear rise at low shear rates was attributed to the elastic yielding process of the paste, while the linear relationship of the Bingham model was manifested in the plastic flow region (at the high shear rate range), in which the shear stress of each group was found to increase steadily and linearly with increasing shear rate, consistent with the typical characteristics of Bingham fluids. The Bingham fluid type of the cement mortar was not altered by the incorporation of the core–shell emulsion, but significant regulatory effects on its yield stress, yield point location, and the extreme value of shear stress were observed. This finding was consistent with the results reported by Chen et al. [14], in which a significant dosage-dependent regulatory effect of the polymer on the rheological parameters of cement mortar was demonstrated. The experimental results were presented in Table 5 and Figure 5.

For the control group, the shear stress of the mortar was 501.1 Pa. After the incorporation of 2% and 4% core–shell emulsion, the shear stress of the mortar was increased to 1448.5 Pa and 1565.3 Pa, respectively, which represented increases of 189.1% and 212.4% compared with the control group, indicating that an order-of-magnitude increase in the yield stress of the mortar was achieved by a low dosage of emulsion, and the structural stability of the mortar under low shear was better at 4% dosage, with plastic flow occurring only after the critical shear rate was reached. It was clarified by the study that 4–6% was the critical dosage range for the regulation of the yield stress of cement mortar by the core–shell emulsion: within the 2–4% dosage range, the increase in yield stress of the mortar was only 8.1%, indicating a limited structural strengthening effect; when the dosage was increased from 4% to 6%, the shear stress of the mortar jumped to 2814.0 Pa, which was an increase of 79.8% compared with the 4% dosage, and a significant structural strengthening mutation was observed; when the dosage was further increased to 8% and 10%, the shear stress of the mortar reached 4099.9 Pa and 5196.6 Pa, respectively, which were increases of 718.2% and 937.0% compared with the control group, and at 10% dosage, the yield stress of the mortar was increased by nearly 10 times compared with the control group. The essence of this phenomenon was identified as the adsorption saturation effect, and the three-dimensional network construction mechanism of the core–shell emulsion in the cement paste, and the core action mechanism was as follows:

(1) The shell layer of the core–shell structured emulsion was composed of hydrophilic active segments carrying carboxyl and hydroxyl groups, while the core layer was composed of a hydrophobic polymer. In the alkaline environment of cement mortar, the emulsion particles were directionally anchored onto the surface of cement particles and hydration products through electrostatic adsorption and hydrogen bonding [15]. When the dosage was lower than 4%, the emulsion particles were only able to partially cover the surface of the cement particles, and a complete cross-linked three-dimensional network was not formed. When the dosage reached 4–6%, saturated adsorption of the emulsion particles on the cement particle surface was achieved, and the excess particles were filled into the interparticle gaps, thereby greatly increasing the cross-linking density of the system. The internal structural strength of the paste was significantly enhanced, and a higher shear stress was required to destroy the network and initiate plastic flow. Macroscopically, this was manifested as an abrupt increase in the yield stress.

(2) The hydrophilic groups on the shell layer of the emulsion were able to absorb a large amount of free water in the paste, thereby reducing the content of free water that served as a lubricant and significantly increasing the internal frictional resistance between cement particles. As the emulsion dosage was increased, the free water absorption effect was continuously enhanced, which synergistically interacted with the three-dimensional network strengthening effect, ultimately leading to a stepwise increase in yield stress with increasing dosage [16,17].

(3) The active groups on the emulsion shell layer were found to undergo a complexation reaction with Ca^2+^ released during cement hydration. On one hand, the early hydration of cement was delayed by this reaction, thereby preventing structural instability caused by the rapid agglomeration of hydration products. On the other hand, the cross-linked structure formed by complexation was shown to enhance the internal cohesion of the paste, which further amplified the yield stress enhancement effect at higher dosages [18,19].

#### 3.1.2. Creep Recovery

**Figure 6:**
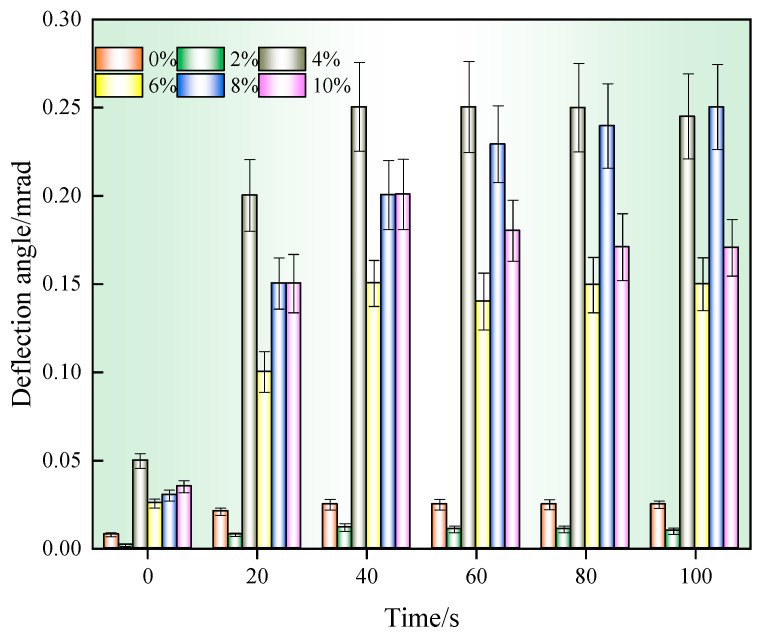
Deflection angle vs. time of cement mortar under different emulsion dosages.

The creep profiles of deflection angle versus time for cement mortar modified with different dosages of core–shell structured emulsion were shown in Figure 6, and the test results were presented in Table 6.

It was observed from Figure 6 that the creep process of all specimens followed the deformation law of viscoelastic materials and could be mainly divided into two characteristic stages. ① Rapid creep stage (0–40s): For the groups with dosages of 4% to 10%, the deformation was mainly concentrated in this interval, and the deformation within 40 s accounted for more than 90% of the total deformation throughout the whole process, corresponding to the instantaneous elastic deformation and structural rearrangement of the internal flocculated network of the paste. ② Steady-state creep stage (40–100s): The deformation rate of all specimens was significantly slowed down, the data profiles tended to become flat, and the deformation was dominated by stable viscous flow without notable structural damage. For the control group, the stable deflection angle at 100 s was 0.024 mrad, and the total deformation increment throughout the whole process was 0.016 mrad. For the 2% dosage group, the stable deflection angle at 100 s was only 0.009 mrad, and the total deformation increment was 0.007 mrad; no obvious rapid creep stage was observed, and the creep deformation resistance was significantly enhanced. When the emulsion dosage reached 4% or above, a jumping increase in the creep deformation of the mortar was exhibited. For the 4% and 8% dosage groups, the stable deflection angles at 100 s reached 0.230 mrad and 0.250 mrad, respectively, which were more than 10 times higher than those of the control group. For the 6% and 10% dosage groups, the deformations were relatively lower, with stable deflection angles at 100 s of 0.145 mrad and 0.166 mrad, respectively, and the creep deformation showed an overall increasing trend with increasing dosage. This phenomenon could be explained as follows.

(1) At a low dosage of 2%, the emulsion particles were directionally anchored onto the surface of cement particles and hydration products through electrostatic adsorption and hydrogen bonding of the active groups on the shell layer. A complete cross-linked three-dimensional flocculated network was thereby constructed, which effectively limited the sliding and structural rearrangement of cement particles under constant stress, increased the proportion of elastic characteristics of the paste, and ultimately resulted in the suppression of creep deformation [20,21].

(2) At a dosage of 4% or higher, saturated adsorption of the emulsion particles on the cement particle surface was reached, and the excess viscous polymer components significantly increased the proportion of viscous characteristics of the paste, thereby shifting the viscoelastic ratio of the paste toward viscous flow dominance. Meanwhile, a lubricating layer was formed between cement particles by the excess emulsion particles, which reduced the internal frictional resistance between particles and further amplified the creep deformation. A competition between the network-strengthening effect and the viscous-lubrication effect of the emulsion was thus induced at high dosages, and consequently, a slight decrease in the creep deflection angle of the mortar was observed in the 6% and 10% dosage groups [22,23,24].

**Figure 7:**
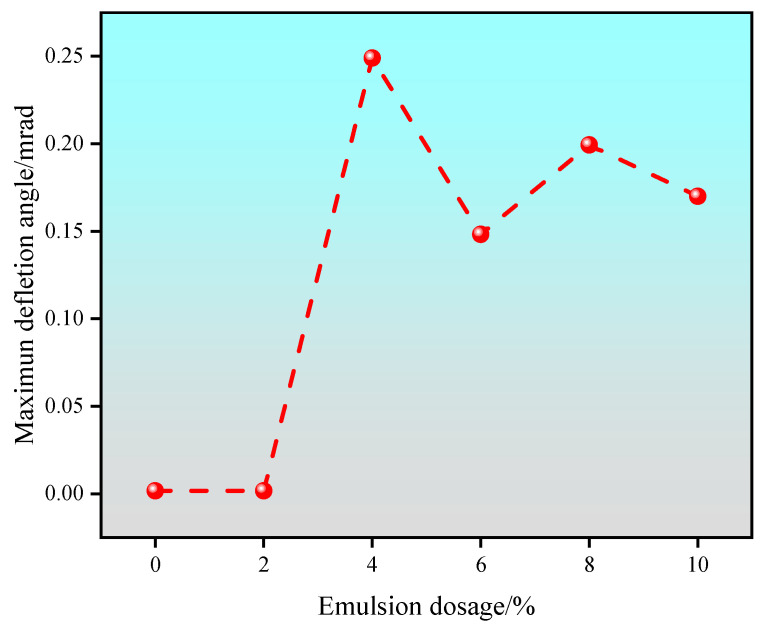
Relationship between maximum deflection angle difference and emulsion dosage.

The difference between the maximum creep deflection angle and the recovered deflection angle was defined as the deflection angle difference. A larger deflection angle difference was associated with a stronger deformation recovery ability of the material; conversely, a smaller deflection angle difference indicated a weaker deformation recovery ability. As can be seen from Figure 7, the deflection angle difference in the core–shell structured emulsion-modified cement mortar did not exhibit a neat and uniform trend, but instead showed a fluctuating jagged development pattern. When the dosage was 0% and 2%, the deflection angle differences in the cement mortar were basically at the same level. However, when the dosage was increased to 4%, 6%, 8%, and 10%, the deflection angle differences in the cement mortar reached nearly 10 times those of ordinary cement mortar. This result strongly indicated that the emulsion was able to significantly enhance the deformation recovery ability of the cement mortar, thereby increasing its elasticity [25].

#### 3.1.3. Dynamic Viscosity

**Figure 8:**
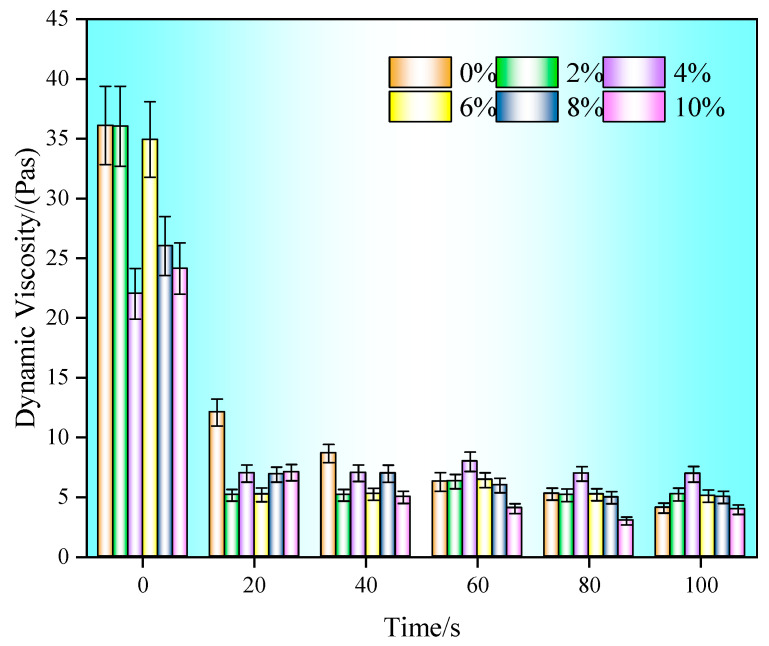
Dynamic viscosity vs. shearing time of cement mortar with different emulsion dosages.

The dynamic viscosity vs. shearing time profiles of cement mortar modified with different dosages of core–shell structured emulsion were shown in Figure 8, and the test results were presented in Table 7.

It was observed from Figure 8 that the dynamic viscosity of each cement mortar specimen decreased with increasing shear time, and the rate of decrease gradually slowed down, i.e., the shear-thinning phenomenon was exhibited [26]. The dynamic viscosity of all specimens showed a typical two-stage variation pattern with increasing shear time. In the rapid decrease stage (0–20 s), this stage was identified as the main reduction interval of dynamic viscosity, where a 62.0% reduction in viscosity was observed for the control group. A higher reduction was shown by the emulsion-modified mortars, and a 76.1% reduction was recorded for the 2% dosage group, indicating a significantly enhanced shear sensitivity. This phenomenon was attributed to the rapid depolymerization of the flocculated network in the paste and the sharp drop in internal frictional resistance between particles under shear. In the stable decay stage (20–100 s), the viscosity decrease rate of all specimens was significantly slowed down, and the data profiles entered a steady-state interval with low-amplitude fluctuations. At 100 s, the viscosity differences among the specimens were greatly reduced, converging within a range of 3–7 Pa·s, indicating that a dynamic equilibrium between shear-induced structural disruption and reconstruction was reached in the paste.

The dosage effect analysis indicated that the initial viscosity of the mortar with a low dosage of 2% was basically comparable to that of the control group, and the initial structural stability of the paste was not compromised. When the dosage was 4% or higher, the initial viscosity of the mortar was significantly reduced overall, except for the 6% dosage group, where no significant difference from the control group was exhibited in terms of initial viscosity, due to the balance between network construction and lubrication effects. More pronounced shear-thinning behavior was exhibited by all modified mortars compared to the control group. At 100 s, the steady-state viscosities of the 2%, 4%, and 6% dosage groups were comparable to that of the control group, while the steady-state viscosities of the high-dosage groups (8% and 10%) were lower, indicating lower flow resistance under sustained shear.

In summary, the regulation effect of the core–shell emulsion on the dynamic viscosity of mortar was essentially attributed to its differentiated regulation of the internal flocculated network and interparticle interactions in the paste, and the mechanism was found to be similar to that observed in the creep recovery tests described above. At a low dosage of 2%, a physically cross-linked three-dimensional flocculated network was constructed by the emulsion particles through electrostatic adsorption and hydrogen bonding. Under static conditions, the network structure ensured the initial viscosity and sag resistance of the mortar, while under shear, rapid depolymerization of the network was induced, resulting in significant shear-thinning behavior. Thus, a perfect balance between static stability and construction flowability was achieved. When the emulsion dosage was 4% or higher, saturated adsorption of emulsion particles on the cement particle surface was reached. The excess particles formed a polymer lubricating layer, which reduced the internal frictional resistance between particles and simultaneously weakened the strength of the initial flocculated network. Consequently, significant reductions in both the initial and steady-state viscosities were ultimately achieved. At a dosage of 6%, a balance was established between the network-strengthening effect and the lubrication-induced viscosity-reduction effect, and therefore no significant decrease in the initial viscosity was observed [26,27,28].

#### 3.1.4. Thixotropy

**Figure 9:**
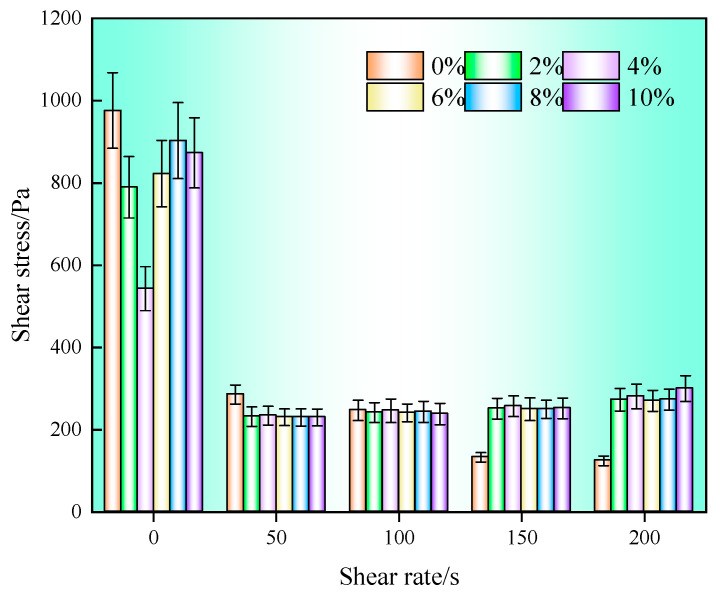
Shear stress vs. shear rate of cement mortar with different emulsion dosages.

A ramp-up and ramp-down cyclic shear mode was adopted for thixotropy measurement, with the shear rate range set from 0 to 200 s^−1^, covering a broader high-shear region. It was specifically used for calculating the thixotropic hysteresis loop area, which was employed to characterize the structural breakdown and recovery behavior of the paste under cyclic shear. The shear stress distributions of cement mortar modified with different dosages of core–shell structured emulsion at shear rates ranging from 0 to 200 s^−1^ are shown in Figure 9 below. Typical shear-thinning behavior was exhibited by all specimens. The highest shear stress was observed at the initial stage, and as the shear rate was increased to 50 s^−1^, a sharp decrease in shear stress (by more than 60%) was exhibited, corresponding to the rapid depolymerization and destruction of the internal three-dimensional flocculated network in the paste. In the shear rate range of 50–200 s^−1^, the shear stress entered a stable fluctuation stage with low amplitude, the differences in shear stress among the groups were greatly reduced, and the flow resistance under high shear tended to be consistent. The test results were presented in Table 8.

It was observed from Figure 9 that when the emulsion dosage was 2% and 4%, the initial shear stress was significantly lower than that of the control group, and the largest reduction in initial stress was observed in the 4% dosage group, indicating that a low dosage of emulsion was effective in reducing the initial structural strength of the mortar and improving its workability. When the dosage was 6–10%, the initial shear stress gradually recovered, and the initial stress of the test groups approached that of the control group, indicating that the network construction effect of a high dosage of emulsion counteracted the lubrication-induced viscosity reduction, thereby restoring the initial structural strength of the paste. In the high-shear stage, no significant differences in shear stress were observed among all dosage groups, indicating that the core–shell emulsion only regulated the initial structure and low-shear response of the mortar without affecting its flowability under high shear. Thus, a perfect optimization of construction performance was achieved, characterized by “thickening/viscosity reduction under static conditions and consistent flowability under shear”.

**Figure 10:**
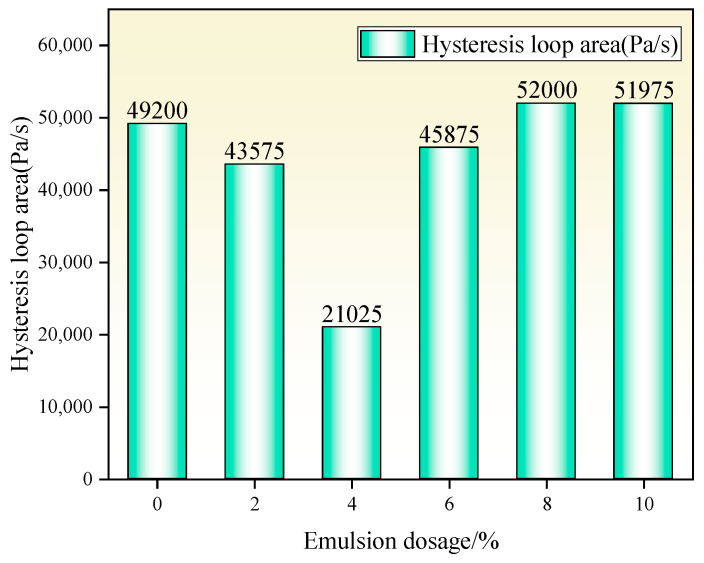
Hysteresis loop area vs. emulsion dosage.

To express the thixotropic strength of the material more intuitively, the hysteresis loop area obtained from the test was integrated according to Formula (4), and the result was rounded to an integer. The result was shown in Figure 10.

A larger area was interpreted as more significant energy dissipation during the structural breakdown and recovery of the paste in the shear cycle, indicating stronger thixotropy. In the low dosage range of 0–4%, the hysteresis loop area was continuously reduced with increasing dosage. The area of the control group was 49,200 Pa/s, and it was reduced to 21,025 Pa/s at a dosage of 4%, representing a 57.3% decrease compared to the control group. Thus, the thixotropy was significantly weakened, and the structural stability of the paste was optimal. In the medium dosage range of 4–8%, the hysteresis loop area rapidly increased. At a dosage of 8%, the area rose to 52,000 Pa/s, which was slightly higher than that of the control group, indicating significantly enhanced thixotropy. In the high dosage range of 8–10%, the hysteresis loop area tended to stabilize. At a dosage of 10%, the area was 51,975 Pa/s, which was basically comparable to that at 8%, and the thixotropy was maintained at a level slightly higher than that of the control group.

The reason was attributed to the fact that the emulsion molecules played roles in filling and bonding, enabling the structure of the cement mortar to be restored to a large extent after being damaged, thereby producing an effect similar to spring recovery. At a low dosage, a stable three-dimensional cross-linked network was constructed by the emulsion particles through adsorption, which reduced structural damage and energy dissipation during the shearing process, thereby weakening the thixotropy. When the dosage exceeded 4%, saturated adsorption of the emulsion particles was reached, and the excess polymer increased the proportion of viscous components in the paste, enhanced the shear hysteresis effect, and caused the thixotropy to rise again. At high dosages, a balance was achieved between the network-strengthening effect and the lubrication effect, and the thixotropy tended to stabilize [29,30,31].

### 3.2. X-Ray Diffraction (XRD)

**Figure 11:**
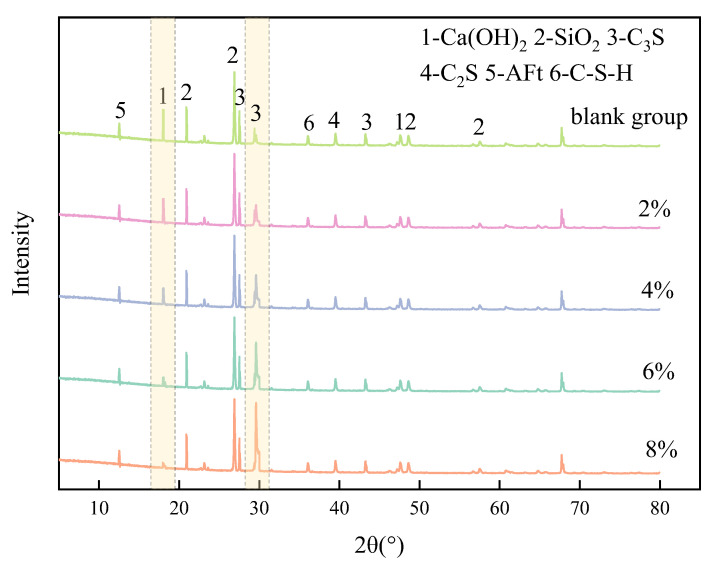
XRD patterns of cement mortar at different emulsion dosages.

The X-ray diffraction patterns of cement mortar with different core–shell emulsion dosages (0, 2%, 4%, 6%, and 8%) after 28 days of curing are shown in Figure 11, and a total of six crystalline phases were identified: CH, AFt, C_3_S, C_2_S, SiO_2_, and C-S-H.

A broadened diffuse background within the 2θ range of 20–35° was exhibited by all specimens, without sharp diffraction peaks, which was a typical characteristic of poorly crystalline calcium silicate hydrate (C-S-H) gel. C-S-H was recognized as a semicrystalline phase with a crystallinity of approximately 15–20%, and it was identified as the most important hydration product of Portland cement [32]. Sharp diffraction peaks were correspondingly attributed to the other five crystalline phases present in the system [33]. Among them, the peak intensity of quartz (26.6°) was found to be basically consistent across all specimens, indicating that quartz was an inherent mineral component of the river sand aggregate and did not significantly interfere with the quantitative analysis of hydration products. With increasing emulsion dosage, the relative contents of the crystalline phases exhibited regular changes: the relative content of the main hydration product, CH, was significantly reduced, indicating that the cement hydration process was inhibited by the emulsion. The relative content of the unhydrated clinker phase C_3_S was significantly increased, which further confirmed that the core–shell emulsion exerted a regulatory effect on cement hydration [4]. No significant differences in the full width at half maximum (FWHM) of the diffraction peaks of the crystalline phases were observed, indicating that the crystallinity of the hydration products was not altered by the emulsion, and only their formation rate and relative content were affected. No new characteristic diffraction peaks were detected, confirming that no chemical reaction occurred between the emulsion and the cement hydration products, and thus no new crystalline phases were generated [34].

In summary, the regulatory mechanism of the core–shell emulsion on cement hydration was clearly revealed by XRD analysis:

(1) Hydration inhibition effect: A positive correlation was found between the emulsion dosage and the relative content of the unhydrated clinker phase, while a negative correlation was observed between the emulsion dosage and the relative content of the main crystalline hydration products. This directly demonstrated that the hydration process of cement at 28 days was significantly inhibited by the emulsion.
(2) Physical barrier-dominated mechanism: The inhibition effect was found to be enhanced with increasing emulsion dosage, and no new phases were detected. This indicated that a polymer film was mainly formed on the surface of cement particles by the emulsion, which thereby hindered the diffusion and transport of water molecules and ions, and consequently, the hydration reaction process was retarded.
(3) Regulation characteristics of phase proportion: The core of emulsion modification was found to lie in the regulation of hydration kinetics and the relative proportions of the phases, rather than in the alteration of phase composition [35]. At a high dosage, the residual unhydrated clinker was considered to provide continuous long-term hydration potential for the mortar, which was beneficial for further densification of the microstructure at later ages.

### 3.3. Fourier Transform Infrared Spectroscopy (FTIR)

**Figure 12:**
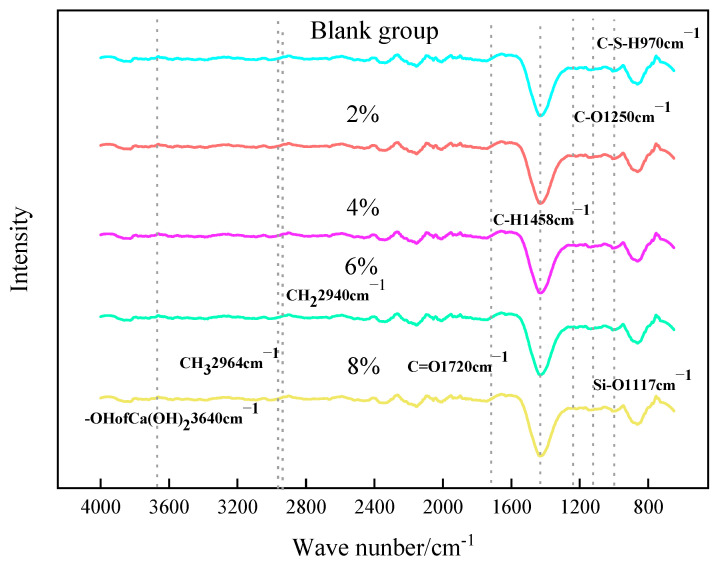
FTIR spectra of cement mortar with different emulsion dosages.

The Fourier transform infrared spectra of the core–shell structured emulsion-modified cement mortar specimens and the blank control group after 28 days of curing are shown in Figure 12. All spectral lines exhibited the typical infrared absorption characteristics of Portland cement hydration products, and the main absorption bands within the wavenumber range of 4000–400 cm^−1^ were clearly attributed to the vibration modes of O–H, C–O, and Si–O, while the characteristic vibrations of organic functional groups introduced by the emulsion were also included [36]. By comparing the spectra of the specimens with emulsion dosages of 2%, 4%, 6%, and 8%, the influence of the emulsion polymer on the cement hydration reaction and the structure of the hydration products was systematically analyzed.

No new characteristic absorption peaks were observed in the spectra of all specimens, indicating that no chemical reaction occurred between the emulsion and the cement hydration products, and no new chemical bonds were formed. This finding mutually confirmed the conclusion from XRD analysis that “no new crystalline phases were generated”, and further supported the view that the cement hydration was influenced by the emulsion through a physical barrier mechanism. The characteristic absorption peak at 3640 cm^−1^ in the spectra was assigned to the stretching vibration of the hydroxyl group (–OH) in calcium hydroxide (CH), while the peak at 2964 cm^−1^ was attributed to the stretching vibration of the methyl group (–CH_3_) in CH. The absorption bands near 2940 cm^−1^ and 1458 cm^−1^ were assigned to the asymmetric stretching vibration of the C–H bond in the organic components of the emulsion. The peak at 1720 cm^−1^ was identified as the stretching vibration of the ester carbonyl group (C=O), which served as a characteristic marker peak of the emulsion polymer. The strong absorption band near 1250 cm^−1^ corresponded to the stretching vibration of the C–O bond, and the absorption band at 1117 cm^−1^ was assigned to the vibration of the Si–O bond. Furthermore, the characteristic peak at around 980 cm^−1^ was directly related to the asymmetric stretching vibration of the Si–O bond in calcium silicate hydrate (C-S-H), the main hydration product of cement, and was thus identified as the characteristic absorption peak of C-S-H gel [37].

In summary, the regulatory effect of the core–shell emulsion on cement hydration was confirmed by FTIR analysis at the molecular level. The emulsion was found to exist in the cement matrix in a physically dispersed form. A polymer film was formed on the surface of cement particles by the emulsion, which hindered the hydration reaction from proceeding. Only the amount of hydration products generated was altered by the emulsion, while their chemical composition and molecular structure were not changed.

### 3.4. Scanning Electron Microscopy (SEM)

**Figure 13:**
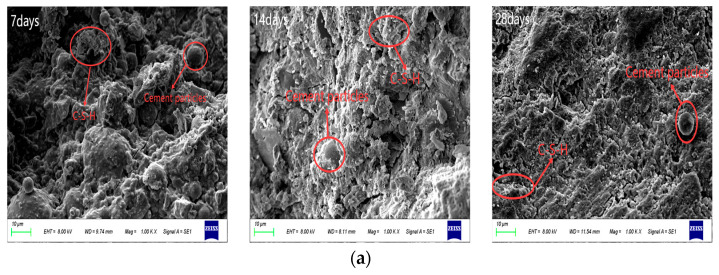

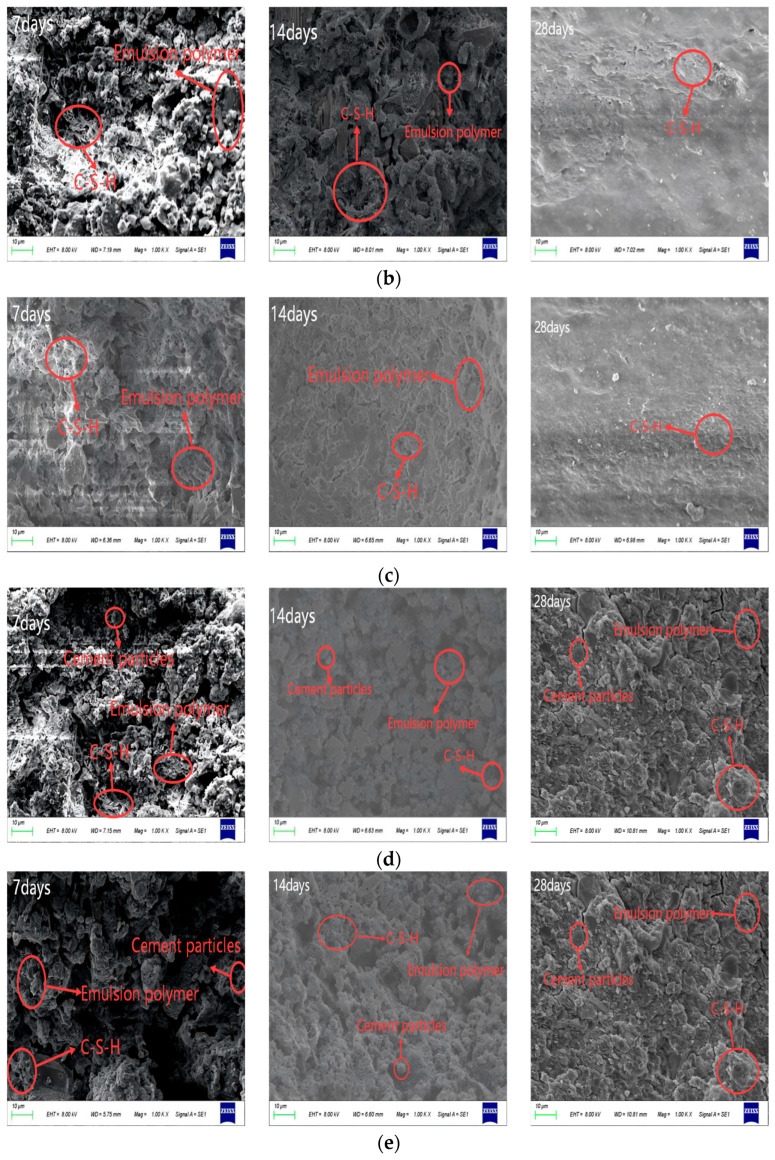
Microscopic morphology of cement mortar at different curing ages with different dosages of core–shell structured emulsion. (**a**) Microscopic morphology of ordinary cement mortar at different curing ages. (**b**) Microscopic morphology of cement mortar with 2% core–shell emulsion dosage at different curing ages. (**c**) Microscopic morphology of cement mortar with 4% core–shell structured emulsion at different curing ages. (**d**) Microscopic morphology of cement mortar with 6% core–shell structured emulsion at different curing ages. (**e**) Microscopic morphology of cement mortar with 8% core–shell structured emulsion at different curing ages.

Scanning electron microscopy (SEM) was recognized as a core method for intuitively characterizing the morphology of hydration products, pore structure, and the interfacial bonding state between the polymer and the cement matrix, serving as a key means to verify the hydration patterns observed by XRD/FTIR and to reveal the microscopic origin of macroscopic rheological properties. In this study, the morphological evolution of cement mortar modified with 0–8% core–shell emulsion at curing ages of 7, 14, and 28 days was comparatively analyzed. Combined with the analysis of hydration product composition described above, the regulation mechanism of the emulsion on the microstructure of cement mortar was elucidated. The micro-morphology of the specimens with different emulsion dosages and at different curing ages is shown in Figure 13.

#### 3.4.1. Evolution of Microscopic Morphology at Different Curing Ages

(1) 7-day early hydration stage

In the control group, cement hydration was vigorous, flake-like CH crystals were extensively stacked, C-S-H gel was loosely distributed, and the matrix was rich in interconnected capillary pores and unhydrated cement particles, resulting in a loose and porous structure. In the group with 2~4% addition, the emulsion physically adsorbed onto cement particles, forming a continuous polymer film, which delayed early hydration. The number and size of CH crystals were significantly reduced, the C-S-H gel was uniformly distributed, pores were filled, and the matrix compactness was notably improved. In the 6~8% addition group, the polymer wrapping effect was further enhanced, resulting in a greater number of unhydrated particles, which was consistent with the increased diffraction peak intensity of C_3_S/C_2_S observed in XRD. Although pores were filled by the polymer, a continuous network was not formed at the early stage, and local agglomeration occurred [38,39].

(2) 14-day intermediate hydration stage

Through the observation of the development of hydration products and the number of residual unhydrated particles in Figure 13a, the degree of hydration was found to be increased in the control group. The CH crystals were observed to grow and align directionally, and a preliminary three-dimensional network was formed by the overlapping of the C-S-H gel. However, a large number of interconnected pores still remained in the matrix, and significant structural defects were exhibited. In the 2–4% dosage groups, the polymer film and the hydration products were found to be intertwined and fused with each other, forming a “cement hydration product-polymer” composite cross-linked network. The CH crystals were wrapped and refined, the pores were further filled, and the compactness of the matrix was greatly enhanced. In the 6–8% dosage groups, a large number of unhydrated particles still remained. However, the polymer was found to have formed a preliminary continuous phase, which filled most of the capillary pores and was able to bridge adjacent cement particles, thereby enhancing the integrity of the matrix. Only a small number of microcracks were observed at the interface between the hydration products and the polymer [40].

(3) 28-day standard curing age

Based on the XRD peak intensities of the unhydrated clinker phases (C_3_S, C_2_S) and the main hydration product, calcium hydroxide (CH), in the control group in Figure 11, as well as the development of hydration products and the number of residual unhydrated particles observed in Figure 13a, the hydration of the control group was inferred to be essentially complete. The CH crystals were found to be coarse and densely stacked, and the C-S-H gel was observed to be flocculently agglomerated, with a considerable number of capillary pores and microcracks still remaining in the matrix. In the 2–4% dosage groups, the composite network was found to be well developed, and a continuous three-dimensional skeleton was formed by the polymer film penetrating through the hydration products. The CH crystals were completely wrapped and refined, and the lowest porosity as well as the most dense and uniform structure were exhibited by the matrix, which was identified as the microstructural origin of the weakest thixotropy and the optimal structural stability of the mortar at 4% dosage. In the 6–8% dosage groups, the degree of hydration was still significantly lower than that of the control group. Although the polymer formed a complete continuous phase and fully filled the pores, the proportion of hydration products was reduced due to the excessive polymer, and polymer-rich zones were formed in the matrix, which reasonably explained the differences in macroscopic rheological properties of the mortar at high dosages [41].

#### 3.4.2. Dosage-Dependent Regulation Effect of Emulsion on Microstructure Evolution

A significant dosage dependence was exhibited by the core–shell emulsion’s regulation of the cement mortar microstructure, which was highly consistent with the analysis results of hydration products described above:

Hydration inhibiting effect: As the emulsion dosage was increased, the polymer coating layer on the surface of cement particles was gradually thickened; thereby, the diffusion and transport of water and ions were hindered, and the cement hydration process was delayed. This was manifested as follows: as the dosage was increased, the number of unhydrated C_3_S/C_2_S particles was increased, the size of CH crystals was reduced, and their content was decreased, which was completely consistent with the variation pattern of diffraction peak intensities in XRD [42,43].

Pore filling and structural densification: At low dosage (2~4%), the emulsion particles were uniformly dispersed in the matrix, capillary pores were filled, cement particles were bridged, and matrix compactness was significantly improved. A dosage of 4% was determined as the optimal dosage, at which polymer dispersibility was the best, no agglomeration was observed, and the structural densification effect was the most significant [44].

Continuous polymer phase formation: After the dosage was increased beyond 4%, a continuous polymer phase was formed by the excess emulsion in the matrix, and the space of some hydration products was replaced; at a dosage of 8%, the polymer phase had become an important component of the matrix; although porosity was further reduced, the viscoelastic proportion of the matrix was shifted toward viscosity dominance [45,46].

#### 3.4.3. Correlation Mechanism Between Microstructure and Macroscopic Rheological Properties

The macroscopic rheological properties of the cement mortar were directly determined by the evolution of the microstructure, and the intrinsic correlation between the two was expressed as follows:

Mechanism of yield stress enhancement: A “cement particle-polymer” composite network was constructed by the emulsion through physical adsorption and bridging action, and the interaction force between particles was enhanced. As the dosage was increased, the strength of the composite network was continuously increased; therefore, the yield stress of the mortar was increased significantly in a stepwise manner.

Mechanism of creep resistance optimization: At 2~4% dosage, the sliding and structural rearrangement of cement particles were effectively limited by the dense composite network; therefore, the creep deformation of the mortar was significantly reduced. After the dosage was increased beyond 4%, the viscous characteristics of the continuous polymer phase were highlighted, by which the paste was made prone to viscous flow under constant stress, and an order-of-magnitude increase in creep deformation was observed.

Mechanism of thixotropy regulation: At 4% dosage, the composite network structure was the most stable; under shear action, the degree of structural breakdown was low, and the recovery rate was fast; therefore, the thixotropic hysteresis loop area was minimized, and the thixotropy was the weakest. At high dosage, significant hysteresis was exhibited by the shear breakdown and recovery of the continuous polymer phase, which caused the thixotropy to increase again and tend to stabilize.

**Figure 14:**
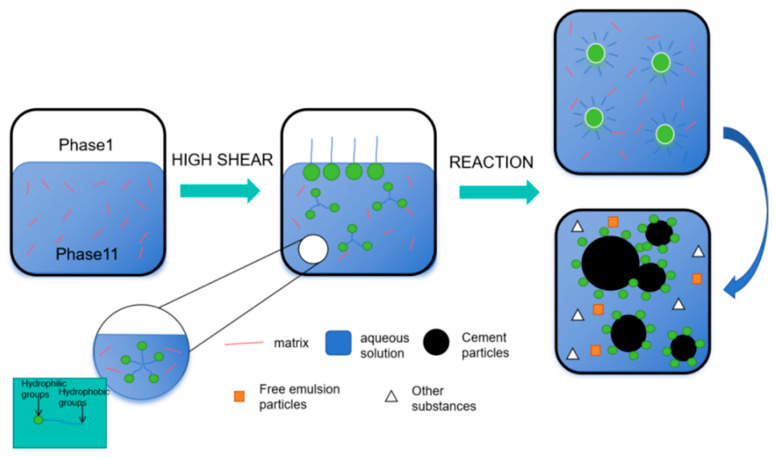
Schematic diagram of the microscopic action mechanism of core–shell emulsion in cement-based materials.

In summary, the microstructure of cement mortar was regulated by the core–shell emulsion through three synergistic effects, namely, hydration retardation by physical coating, pore filling, and densification, and composite network enhancement [47]. Consequently, precise control over the macroscopic rheological properties was achieved, and a microstructural theoretical basis was provided for its engineering application. The microscopic action mechanism of the emulsion in cement mortar was illustrated in Figure 14, in which the adsorption state of emulsion particles on the surface of cement particles, as well as the structural evolution behavior under static and high-shear conditions, was shown.

## 4. Conclusions

In this study, a core–shell structured emulsion admixture was prepared by free radical polymerization. The regulatory effect of its dosage (ranging from 0% to 10%) on the rheological properties of cement mortar was systematically investigated, and the underlying mechanism was elucidated by combining multi-scale microstructural characterizations, including XRD, FTIR, and SEM. The main conclusions were as follows:

1. The Bingham fluid behavior of cement mortar was not altered by the core–shell emulsion, whereas a significant dosage-dependent regulatory effect on yield stress was observed, with a critical regulation interval of 4% to 6% being identified. This effect was attributed to the saturated adsorption behavior of emulsion particles on the surface of cement particles. After saturated adsorption was reached, the crosslinking density of the system was significantly increased, and the structural strength of the paste was synergistically enhanced by the combined effect of free water adsorption.
2. Nonlinear regulatory effects on the creep recovery, dynamic viscosity, and thixotropy of the mortar were exhibited by the emulsion. At dosages ranging from 2% to 4%, synergistic optimization of the static structural stability and construction flowability of the mortar was achieved, resulting in a performance balance characterized by “stability at rest and easy flow under shear”. Consequently, this dosage range was identified as the optimal range for balancing construction workability and structural stability.
3. It was confirmed by microstructural analysis that no chemical reaction occurred between the core–shell emulsion and the cement hydration products. The cement hydration process and microstructural evolution were regulated by three physical effects, namely, hydration retardation by physical coating, pore filling and densification, and composite network enhancement. Through these mechanisms, an intrinsic correlation between macroscopic rheological parameters and microstructural evolution was established.
4. The mechanism by which the core–shell structured emulsion regulated the rheological properties of cement mortar was revealed in this study, and the optimal dosage range for engineering applications was identified. A theoretical basis and technical support were thereby provided for the promotion and application of core–shell polymer admixtures in high-performance cement-based materials. Further studies on the combined effects of the emulsion with commonly used admixtures, as well as its influence on the long-term mechanical and durability performance of mortar, could be conducted in the future.

## Figures and Tables

**Table 1:** Chemical and Mineral Compositions of Cement (%).

Composition	CaO	SiO_2_	Fe_2_O_3_	Al_2_O_3_	SO_3_	MgO	f-CaO	C_3_S	C_2_S	C_3_A	C_4_AF
Content (%)	65.78	21.79	2.67	4.59	1.67	1.56	0.88	60.67	17.34	7.67	8.56

**Table 2:** Physical and Mechanical Properties of Cement.

Specific Surface Area (cm^2^/g)	Flexural Strength/MPa		Compressive Strength/MPa		Setting Time/Min	
3.2	3 d	28 d	3 d	28 d	Initial setting	Final coagulation
	5.3	8.5	14.4	48.5	136	190

**Table 3:** Fine Aggregate Sieve Analysis Results.

Sieve Hole (mm)	0.6	0.3	0.15	0.075	Sieve Bottom
Subtotal sieve residue (%)	15.4	42.3	37.8	2.8	1.7

**Table 4:** Mix Proportion of Cement Mortar.

No.	Water (g)	Cement (g)	Sand (g)	Emulsion (g)	Emulsion Dosage (wt.%)	Water-to-Cement Ratio (wt.%)	Sand-to-Cement Ratio (wt.%)
1	225	450	1350	0	0	50	300
2	225	450	1350	9	2	50	300
3	225	450	1350	18	4	50	300
4	225	450	1350	27	6	50	300
5	225	450	1350	36	8	50	300
6	225	450	1350	45	10	50	300

**Table 5:** Shear stress of mortar with different dosages at various shear rates (Unit: Pa).

Shear Rate/s	Blank Control	2% Emulsion Dosage	4% Emulsion Dosage	6% Emulsion Dosage	8% Emulsion Dosage	10% Emulsion Dosage
0.002	84.5	121.6	16.2	35.3	307.2	213.6
0.004	386.1	1196.8	207.3	196.4	2803.5	3208.8
0.006	429.5	1306.6	1503.7	2502.1	3706.6	4515.9
0.008	501.1	1396	1525	2706.8	3906.8	5005.3
0.01	499.2	1448.5	1565.3	2814	4099.9	5196.6

**Table 6:** Creep deflection angle of mortar at various dosages at different times (Unit: mrad).

Time (s)	Blank Control	2% Emulsion Dosage	4% Emulsion Dosage	6% Emulsion Dosage	8% Emulsion Dosage	10% Emulsion Dosage
0	0.007	0.002	0.05	0.025	0.031	0.035
20	0.021	0.008	0.2	0.101	0.148	0.151
40	0.025	0.012	0.25	0.151	0.201	0.201
60	0.024	0.011	0.245	0.125	0.229	0.179
80	0.024	0.011	0.24	0.149	0.239	0.168
100	0.023	0.009	0.23	0.145	0.250	0.166

**Table 7:** Dynamic viscosity of mortar with different dosages at different times (Unit: Pa·s).

Time (s)	Blank Control	2% Emulsion Dosage	4% Emulsion Dosage	6% Emulsion Dosage	8% Emulsion Dosage	10% Emulsion Dosage
0	33.7	33.9	22.1	32.9	26.1	24.1
20	12.8	7.2	6.9	7	6.9	7.1
40	10.1	7.1	7	6.9	6.7	5.1
60	7.6	7.7	7.9	7.8	5.9	4
80	7.2	7.1	6.9	6.9	5.1	3.1
100	6.1	7.0	6.8	6.8	4.9	3.9

**Table 8:** Shear stress of mortars with various dosages under different shear rates (Unit: Pa).

Time (s)	Blank Control	2% Emulsion Dosage	4% Emulsion Dosage	6% Emulsion Dosage	8% Emulsion Dosage	10% Emulsion Dosage
0	976.1	789.7	498.1	822.7	903.2	873.4
50	285.8	232.1	234.5	230.7	230.1	229.9
100	247.2	241.7	246.4	241.3	243.6	238.4
150	133.3	251.4	257.1	250.1	249.9	252.1
200	124.4	272.9	280.8	270.4	273.2	300.1

## Data Availability

The original contributions presented in this study are included in the article. Further inquiries can be directed to the corresponding author.

## Abbreviations

The following abbreviations are used in this manuscript:

SEM	Scanning Electron Microscope
XRD	X-ray Diffractometer
FT-IR	Fourier Transform Infrared Spectrometer
C-S-H	Calcium Silicate Hydrate
AFt	Ettringite
CH/Ca(OH)_2_	Calcium Hydroxide
